# Turtles for Sale: Species Prevalence in the Pet Trade in Poland and Potential Introduction Risks

**DOI:** 10.3390/ani15182711

**Published:** 2025-09-16

**Authors:** Jakub Badziukiewicz, Milena Bors, Rafał Maciaszek, Wiesław Świderek

**Affiliations:** Department of Animal Genetics and Conservation, Institute of Animal Sciences, Warsaw University of Life Sciences, Ciszewskiego 8, 02-786 Warsaw, Poland

**Keywords:** Invasive Alien Species (IAS), biodiversity, *Pseudemys*, *Mauremys reevesii*

## Abstract

The popularity of exotic animals, including turtles, kept as pets, has grown steadily in recent decades. Limited awareness of the biological and ecological requirements of these species, combined with insufficient regulation in the pet trade, has contributed to frequent cases of release into the wild. Consequently, the risk of the introduction of non-native turtle species has increased; while not all introduced species establish viable populations or compete with native fauna, some compete for habitat and food resources, transmit pathogens and parasites, or disrupt ecosystem functioning. This study assessed the availability and prevalence of problematic turtle species in the Polish pet trade. Data were collected in 2024 from three main distribution channels: zoological fairs, pet shops, and online platforms, resulting in 1001 recorded observations. A total of 15 freshwater and 11 terrestrial turtle species were identified. The most frequently offered were members of the genus *Pseudemys* (49.5%) and *Mauremys reevesii* (13.84%)—both potentially invasive taxa already recorded in natural environments of several European countries. Additionally, species prohibited from trade in Poland, such as the pond slider *Trachemys scripta* and the false map turtle *Graptemys pseudogeographica*, were found among online listings, constituting a total of 3.03% of all observations. These results highlight a significant risk of further introduction of alien turtles into Poland’s natural environments, posing a serious threat to biodiversity, including the endangered native European pond turtle *Emys orbicularis*. Urgent public education and additional legal regulations are needed for the pet trade to prevent the further spread of invasive turtle species in Poland and across Europe.

## Simple Summary

The popularity of exotic animals, including turtles, kept as pets, has grown steadily in recent decades. Many species grow large, live for decades, and require complex care. When owners cannot meet these needs, turtles are often released into the wild. Some of these species can become invasive alien species that threaten native biodiversity. This study examined the turtle trade in Poland during 2024 by monitoring zoological fairs, pet shops, and online platforms. In total, 1001 offers were recorded, representing 26 species. The most frequently offered were members of the genus *Pseudemys* (49.5%) and *Mauremys reevesii* (13.84%). Both are already found in the wild in Europe. Additionally, 3.03% of the offers included species prohibited from trade in Poland, such as the pond slider (*Trachemys scripta*) and the false map turtle (*Graptemys pseudogeographica*). Mislabeling of species was frequent, especially in online listings. These findings demonstrate that the Polish pet trade creates a pathway for further introductions of alien turtles into the wild.

## 1. Introduction

The global pet trade facilitates the movement of exotic species, often resulting in unforeseen ecological consequences when these species establish themselves in non-native habitats [1,2,3]. In 2023, the exotic pet trade in North America and Europe was valued at USD 1.3 billion [4]. It has been steadily growing in the past few decades and is expected to keep expanding. The reptile trade, in particular, is projected to grow fastest, with an 8% compound annual growth rate [4]. This increased interest in reptile husbandry may be attributed to evolving consumer preferences, advances in veterinary care, and greater accessibility to reptiles and related care products [5].

The idea of reptiles being easy to keep as pets remains widespread, posing significant challenges [6,7]. Reptiles are often seen as low-maintenance compared to mammals. In reality, they spend most of their lives in enclosures that must be large enough for natural posture and behaviour [8]. Each species has specific microclimate needs, often requiring complex and costly equipment. Diet also varies with species, sex, age, and condition—no universal commercial feeds exist. If all of the above requirements are not met, the reptile may experience health issues that require veterinary intervention. Veterinarians specializing in exotic pets are rarer than those treating domestic or farm animals, making them less accessible and often more expensive [6,7]. Considering all of this, it cannot be said that reptiles are easy to maintain. Although some species are more tolerant than others, they still require time, funds, and most importantly, willingness to learn and adjust to their needs [8].

Turtles are among the most popular reptiles in the pet trade. According to the 2024 Convention on International Trade in Endangered Species of Wild Fauna and Flora (CITES) report, the number of individual turtles amounted to 63,213 individuals [9]. These are just turtles of species covered by the CITES Appendices; the actual total traded in 2024 is significantly higher.

Turtles are long-lived animals, usually bought young and small. Their longevity and the maximum size of some species may contribute to the practice of turtle release in non-native habitats [10,11]. These turtles lack proper care. In some cases, they may establish themselves outside their native range and become categorized as Invasive Alien Species (IAS) [12]. Invasive Alien Species (IAS) are non-native organisms introduced by humans, intentionally or unintentionally, that may harm native biodiversity, ecosystem services, and human well-being [12]. In some regions, such as the European Union, specific legislation [13] governs the prevention and management of these species, but similar concerns apply globally. Due to current invasions or invasion risks, Poland prohibits keeping, breeding, and importing four turtle species. The pond slider (*Trachemys scripta* [14]), native to southeastern and central United States and northern Mexico, is regulated as an IAS of Union concern under the European Union Regulation (EU) No 1143/2014, which applies in all Member States, including Poland. The common snapping turtle (*Chelydra serpentina* [15]), the painted turtle (*Chrysemys picta* [16]), and the false map turtle (*Graptemys pseudogeographica* [17]), all native to North America, are listed as IAS of Member State concern specifically under Polish law—the Act on Alien Species [18]—which implements and complements the EU regulation by defining species restrictions within Poland. Once established, invasive populations are difficult to eradicate [19]; therefore, preventive measures are essential to identify problematic species.

In response to the above, this study aims to assess the prevalence of turtle species in the Polish pet trade. By examining various trade channels and factors influencing pet turtle abandonment, we aim to identify potentially problematic turtle species. The intended outcome is to inform which species should be studied for their establishment risk in Poland.

## 2. Materials and Methods

Data were gathered from three primary trade channels: zoological fairs, pet shops, and online platforms. Observations took place in March, June, September, and December 2024, focusing on identifying and quantifying turtle species offered for sale or adoption. Three of the largest zoological fairs in Poland were selected: one wholesale fair in Łódź and two retail fairs in Łódź and Kraków. Additionally, 29 pet shops in Łódź (8), Kraków (12), and Warsaw (9) were surveyed once in March, June, and September 2024. Exact addresses of pet shops and fairs were omitted due to legal restrictions. During each visit, experts recorded the number and species of turtles available, assessed the species labelling accuracy, and documented any relevant welfare conditions. The welfare assessment was based on expert judgment by a specialist with experience in the welfare of captive reptiles, while no formal scoring system was applied, the evaluation followed the minimum standards outlined in Polish animal protection regulations and broadly accepted reptile care guidelines. Simultaneously, online listings, by private individuals, commercial sellers, and adoption offers were reviewed and systematically monitored in March, June, September, and December 2024, with only active listings during these periods recorded [Table 1]. Online listings were categorized as private individual offers (online private offers) and online pet shop offers (online company offers). Duplicate listings within the same platform and quarter were filtered to avoid redundancy; however, repeated listings reappearing in subsequent quarters were retained, as they may indicate long-term availability and reduced saleability of older turtles. Cross-platform duplication was not observed. All listings were reviewed manually, and care was taken to assign each to the appropriate seller category.

Listings without photos or insufficient data to confirm identification to at least the genus level were excluded from analysis. At zoological fairs and pet shops, the following data were noted: species offered, number of individuals, labelling or species description used by the seller, and seller type (only for fairs; omitted in shops due to consistently commercial context).

All data were entered into a Microsoft Excel database to calculate species occurrence frequency. IBM SPSS Statistics 31.0.0.0 was used to perform chi-square tests for each source in order to determine whether significant differences among offered species were present. Because the exact number of individual turtles in online offers could not be determined, each such observation corresponds to a single offer, not an individual turtle. Turtles were identified to species level when possible, or to genus level if ambiguous (e.g., genus *Pseudemys* due to contentious taxonomy). Experienced herpetologists identified turtles based on morphological traits such as shell shape, plastron colouration, and head pattern. When necessary, trade descriptions and photographs supported species determination.

## 3. Results

**Figure 1:**
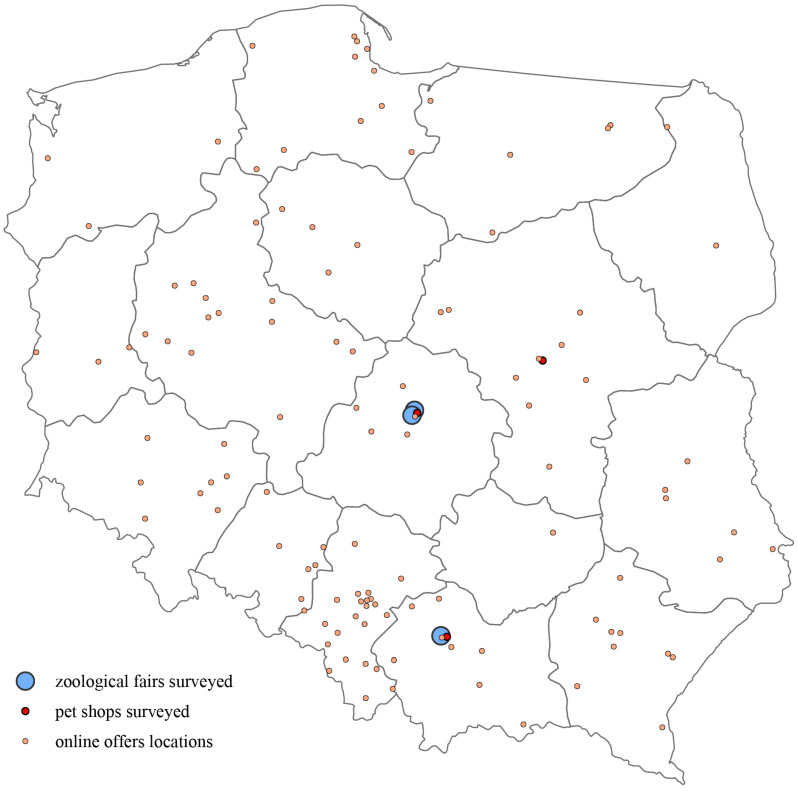
Surveyed trade locations for turtles in Poland through different sources, including zoological fairs, pet shops, and online platforms.

A total of 1001 turtle observations were recorded across all sources [Table 2] [Figure 1].

The dataset included 15 freshwater turtle species and 11 tortoise species (land turtles). The distinction between these ecological groups is critical for understanding market dynamics, as aquatic species dominate trade volume, while tortoises hold a smaller but relevant share. Zoological fairs accounted for the highest number of observations (n=559), followed by online private listings (n=237), online company offers (n=100), and pet shops (n=94). The exact numbers per source and species are detailed in Table 2. Species identification was confirmed based on available listing data, including photographs and vernacular names. However, eleven online listings lacked sufficient descriptive content, such as images or scientific names, to allow for reliable identification to the species or genus level, while these entries were excluded from species-level taxonomic analysis (hence the total number of turtle count of 990), they were retained for labelling accuracy assessment and included in the mislabelling rate in Table 2.

**Figure 2:**
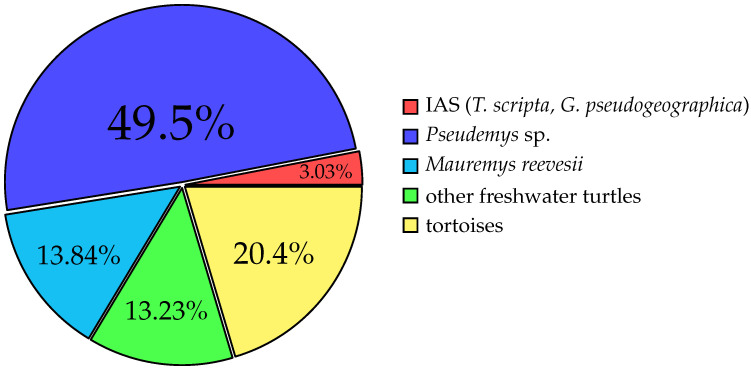
Composition of turtles in the pet trade, including tortoises and Invasive Alien Species regulated in Poland (e.g., *Graptemys pseudogeographica*) and the EU (*Trachemys scripta*).

Among all recorded turtles, the most frequently offered belonged to the genus *Pseudemys* (49.5%), dominating both online and fair sales. The Chinese pond turtle (*Mauremys reevesii*) was the second most common species, constituting 13.84% of the total. Other species were significantly less represented. The percentages of species and genera of interest due to the risk of establishing invasive populations (*Pseudemys*, *Mauremys reevesii*), based on reports from other European countries and species illegal in Poland’s pet trade, are highlighted in Figure 2. The graph shows the dominance of the genus *Pseudemys*, as well as the presence of illegally owned turtle species.

The pie chart [Figure 2] illustrates the proportional distribution of the most represented taxa. It also highlights species of regulatory concern, such as *Trachemys scripta*, banned in the EU, and *Graptemys pseudogeographica*, regulated nationally in Poland. Chi-square goodness-of-fit tests showed significant deviations from theoretical distribution for every source (fairs: $\chi^{2}=66,308$, df=9, p<0.001; pet shops: $\chi^{2}=65,000$, df=6, p<0.001; online private: $\chi^{2}=27,077$, df=14, p=0.019; online companies: $\chi^{2}=21,385$, df=7, p=0.003). A slight preference for freshwater turtles over tortoises was observed in pet shops. The most common species were the Chinese pond turtle *Mauremys reevesii* and the Russian tortoise *Testudo horsfieldii*. At zoological fairs, freshwater turtles were overwhelmingly preferred over tortoises, with the genus *Pseudemys* dominating. Online offers also showed a preference for freshwater turtles, particularly *Pseudemys* species. Across all trade channels, species misidentification or incomplete labelling was a notable issue. Errors were most frequent in private online listings (39%), followed by company listings (31%) and pet shops (20%). Zoological fairs had the lowest error rate (13%).

## 4. Discussion

This study presents the first comprehensive, multi-channel analysis of the turtle pet trade in Poland, combining data from zoological fairs, pet shops, and online listings. By including both freshwater and terrestrial turtle species, the research provides a broad and realistic picture of the market. Notably, it highlights the dominance of freshwater species, particularly those of the genus *Pseudemys*, and draws attention to the continued illegal trade in restricted species such as *Trachemys scripta*. These findings contribute insight into trade patterns, species prevalence, and potential invasion risks in Central Europe. Zoological fairs had the lowest labelling error rate, likely due to the presence of more experienced vendors. Mislabelling most often involved generic terms like “freshwater turtle” or incorrect common names. Unsurprisingly, the most frequently mislabelled turtles in private listings were the banned pond sliders *Trachemys scripta*, despite being prohibited from trade in Poland under Regulation (EU) No 1143/2014.

The most represented turtles were those of the genus *Pseudemys*, native to North America. Due to the contentious taxonomy of the genus, we did not identify these turtles to the species level. Molecular studies of *Pseudemys* found little to no evidence supporting the delimitation of the previously established nine *Pseudemys* species and subspecies [40,41]. Jackson et al. suggest that historical and ongoing hybridisation of *Pseudemys* populations in North America contributes to these findings. Only *Pseudemys gorzugi* and *Pseudemys texana* appear to form monophyletic taxa [40]. This taxonomic uncertainty complicates invasion risk assessments. As the range of *Pseudemys* spans from southeastern New Mexico eastward throughout the Florida peninsula and as far north as Massachusetts, this genus represents an ecologically diverse group. Therefore, it is difficult to precisely assess their habitat requirements and potential for establishment outside their natural range. Any future legislation concerning the trade of *Pseudemys* would need to take into account the challenges of species delimitation and identification. It appears that *Pseudemys* turtles have become the exotic pet trade’s replacement for *Trachemys scripta*, now serving as the most popular freshwater turtle. These turtles are frequently misidentified, poorly regulated, and biologically similar to sliders. However, they may pose an even greater abandonment risk due to their size (up to 40 cm), weight (up to 15 kg), and demanding care requirements [Table 3]. *Pseudemys* turtles have already been introduced in several European countries, including Italy and Portugal [42,43]. In Poland, reports of *Pseudemys* spp. intentionally or unintentionally released into the wild are becoming increasingly frequent [44,45,46].

*Mauremys reevesii*, a species native to East Asia [47], is another emerging risk in the European pet trade. It has already been introduced into the wild in Spain and is increasingly being reported in Poland [48]. Its rising popularity in commercial offers, especially from online private sellers, suggests a growing need to monitor the potential risk of its introduction into wild habitats. Although *M. reevesii* may not reach the size or lifespan of *Trachemys scripta* or *Pseudemys* spp. [Table 3], it is not exempt from the risk of intentional or unintentional release into the wild.

*Trachemys scripta*, native to the southeastern United States and northern Mexico [49], is one of the most widespread freshwater turtle species in the global pet trade. Commonly known as the red-eared slider, it has been introduced to numerous ecosystems worldwide, often outcompeting native turtle species. Despite being listed as an Invasive Alien Species of Union concern under Regulation (EU) No 1143/2014, and therefore illegal to own, breed, and trade in Poland, we recorded a considerable number of online private listings advertising this species. Many such listings avoid direct naming by using vague labels such as “freshwater turtle.” *Trachemys scripta* has long been recognised as one of the world’s most invasive reptiles [50], and despite legal restrictions, it continues to be both traded and observed in the wild in Poland [51,52,53].

*Graptemys pseudogeographica*, native to North America, typically inhabits vegetated rivers and tributaries of the Mississippi and Missouri River basins. Although smaller than *Trachemys scripta* or *Pseudemys*, its environmental sensitivity and behavioural traits, such as aggression during basking [54], can make it a problematic pet. *Graptemys pseudogeographica* is already considered invasive in some regions and is included in the EU IAS list due to its ecological competitiveness and establishment potential.

The risk of establishment of non-native turtle species such as those of the genus *Pseudemys* or *Mauremys reevesii* warrants a separate study, given their prevalence in the pet trade. Similar assessments have been carried out for *Trachemys scripta* and *Graptemys pseudogoegraphica* [44,55]; while *Graptemys pseudogeographica* is encountered infrequently in Polish natural habitats (less frequently than *Pseudemys* spp.), it has been deemed invasive due to its biological and ecological similarity to *Trachemys scripta* [55]. The introduced range of *Trachemys scripta* encompasses the entirety of Poland, primarily due to abandonment into natural habitats by pet owners [44]. No reproductive success has been recorded to date, although experts do not exclude the possibility of *T. scripta* breeding in the Polish climate [44]. *Trachemys scripta* has a considerable impact on natural habitats through predation on amphibians, fish, and invertebrates, as well as through competition with native species.

Only one extant turtle species is native to Poland—the European pond turtle *Emys orbicularis*. According to the International Union for Conservation of Nature (IUCN) Red List, this species is globally classified as Near Threatened (NT). In Poland; however, *E. orbicularis* is considered endangered (EN) and is subject to active conservation measures under both national and European legislation [56]. This species rarely reaches a shell length of 20 cm and is increasingly at risk of being outcompeted by larger, non-native turtles such as *Trachemys scripta*. Under experimental conditions, *T. scripta* has been shown to monopolise basking spots and display aggression towards smaller *E. orbicularis* [57]; while further research is necessary to assess the ecological impact of *Pseudemys* spp. and *Mauremys reevesii* on Polish biodiversity, these alien species may pose significant competitive threats to *E. orbicularis*.

One of the most alarming findings is the role of alien turtles as vectors of pathogens that can affect native wildlife [58,59,60]. In Poleski National Park, hatchling mortality in the European pond turtle was associated with *Chlamydia* infections likely introduced by invasive turtles such as *Trachemys scripta* [44]. Additionally, studies have found potentially zoonotic bacteria such as *Citrobacter* spp. on the shells and in the enclosures of pet turtles, particularly those of *Pseudemys* spp. [61]. The epidemiological risks associated with alien turtles remain understudied and should be considered a critical area for future research. It is also important to note that the rise in temperature associated with climate change, expected to range from 1 to 2 °C, may significantly increase the reproductive success of *Trachemys scripta* and the risk of establishment of other non-native turtle [44]. Despite their ecological and epidemiological risks, *Pseudemys* spp. are not currently listed in national or EU-level IAS registers. This regulatory gap contributes to their popularity and highlights the need for legislative harmonisation. The introduction of a standardised Polish name (e.g., “żółw hieroglifowy” or “hieroglyphic turtle”) could improve public communication and aid enforcement. Likewise, *Mauremys reevesii* should be closely monitored and assessed for potential regulation based on its risk of introduction.

Although the study focused primarily on freshwater turtles, pet tortoises can also be intentionally or unintentionally released into the wild. According to the Global Register of Introduced and Invasive Species, *Testudo graeca* has been introduced in France (two recorded occurrences) and in Poland (one recorded occurrence). However, these cases are significantly less frequent than freshwater turtle introductions and, to date, have not demonstrated measurable ecological impacts [62]. Tortoises are generally considered to have low invasive potential due to their low intentional or unintentional release into the wild rates and limited adaptability to temperate climates [63]. Nonetheless, ongoing climate change may increase habitat suitability in Central Europe for non-native reptile species, including tortoises.

This study has some limitations that should be considered when interpreting the findings. The data represent a partial coverage of the Polish turtle trade, based on selected zoological fairs, pet shops, and online platforms, and therefore do not include all possible outlets. In addition, the relative coverage of each trade channel is not precisely known, which means that direct statistical comparisons between them should be treated with caution. Finally, the analysis was based on a single year, which provides only a cross-sectional perspective; seasonal or long-term changes in trade dynamics may require further study. Broader and repeated surveys would help to build a more comprehensive picture of the turtle trade in Poland.

## 5. Conclusions

The widespread presence of *Pseudemys* spp., *Mauremys reevesii*, and *Trachemys scripta* in the Polish pet trade highlights the risk of further introductions into the wild; while *T. scripta* remains the most widely distributed alien turtle, *Pseudemys* spp. may pose an even greater risk of abandonment. *M. reevesii*, although less frequently recorded, is emerging as a species of concern. These species may threaten the native European pond turtle through competition, habitat displacement, and pathogen transmission. Documented *Emys orbicularis* hatchling mortality linked to *Chlamydia* infections and the presence of zoonotic bacterial loads underscores the importance of addressing the epidemiological dimension of alien species introductions.

To mitigate these risks, we strongly recommend the implementation of monitoring and restriction frameworks as well as the promotion of standardised species naming to aid enforcement and increase public awareness.

## Figures and Tables

**Table 1:** Variables collected from online turtle trade listings and their descriptions.

Data Collected	Description	Unit/Data Type	Possible Options/Notes
Website address	URL of the website where the listing was found	Text (URL)	
Archived offer file name	File name of the saved listing archive	Text	
Date of finding and posting	Date when the listing was found and posted	Date (YYYY-MM-DD)	
Listing status	Whether the listing is currently active or inactive	Categorical	Active/Inactive
Vernacular (common) name used	Common name of the turtle used in the listing	Text	
Scientific name used	Scientific name used in the listing	Text	
Corrected scientific name	Corrected/validated scientific name	Text	
Number of individuals	Number of turtles offered	Integer	
Specimen type	Age or life stage of specimens offered	Categorical	Juvenile/Adult/ Unspecified
Distinctive traits	Any notable features or morphs described	Text	
Unit type	Type of sales unit (per individual, per group, etc.)	Text	Individual/Group
Price per unit (PLN)	Price per unit in Polish zloty	Numeric (PLN)	
Additional comments	Extra notes provided by seller	Text	
Sale conditions	Conditions or terms of sale	Text	
Seller type	Type of seller	Categorical	Private individual/ Commercial seller
Seller name or company name	Name of seller or company	Text	
E-mail address	Contact email	Text	
Phone number	Contact phone number	Text	
Voivodeship/Region	Seller’s region or voivodeship	Text	
Other contact details	Additional contact information	Text	
Notes from direct contact	Notes from any direct communication with the seller	Text	

**Table 2:** Observations of turtle species by trade source, species type, and labelling accuracy.

Species or Higher Taxa	English Name	Total Observations	Fairs	Pet Shops	Online Private	Online Companies
Freshwater turtles		788	517	58	149	64
*Pseudemys* sp. [20]	Pseudemys, cooter, hieroglyphic turtle *	490	394	14	58	24
*Mauremys reevesii* [17]	Chinese pond turtle	137	63	30	29	15
*Mauremys sinensis* [21]	Chinese stripe-necked turtle	70	28	12	15	15
*Trachemys scripta* [14]	Pond slider	27	0	0	27	0
*Trachemys venusta* [20]	Meso-American slider	2	0	0	1	1
*Sternotherus odoratus* [22]	Common musk turtle	22	8	0	11	3
*Graptemys pseudogeographica* [17]	False map turtle	3	0	0	2	1
*Graptemys* sp. [23]	Map turtles	1	0	0	1	0
*Pelomedusa subrufa* [24,25]	African helmeted turtle	3	0	1	1	1
*Pelomedusa* sp. [26]	Pelomedusa	27	24	0	1	2
*Pelusios castaneus* [27]	West African mud turtle	2	0	1	1	0
*Claudius angustatus* [28]	Narrow-bridged musk turtle	1	0	0	0	1
*Pelodiscus sinensis* [29]	Chinese softshell turtle	1	0	0	1	0
*Apalone ferox* [16]	Florida softshell turtle	1	0	0	1	0
*Emydura subglobosa* [30]	Red-bellied short-necked turtle	1	0	0	0	1
Tortoises		202	42	36	88	36
*Malacochersus tornieri* [31]	Pancake tortoise	3	0	0	3	0
*Kinixys belliana* [32]	Bell’s hinge-back tortoise	2	0	0	2	0
*Centrochelys sulcata* [33]	African spurred tortoise	4	0	0	4	0
*Stigmochelys pardalis* [34]	Leopard tortoise	9	2	1	6	0
*Testudo horsfieldii* [35]	Russian tortoise	70	11	24	20	15
*Testudo hermanni* [36]	Hermann’s tortoise	55	12	11	25	7
*Testudo marginata* [37]	Marginated tortoise	26	12	0	11	3
*Testudo graeca* [15]	Greek tortoise	14	2	0	9	3
*Testudo* sp. [15]	Mediterranean tortoises	12	0	0	7	5
*Chelonoidis carbonarius* [38]	Red-footed tortoise	3	0	0	0	3
*Chelonoidis* sp. [39]	Chelonoidis	4	3	0	1	0
All turtles and tortoises		990	559	94	237	100
Source		Total observations	Fairs	Pet shops	Online private	Online companies
		N %	N %	N %	N %	N %
Number of improperly labelled offers		218 22	70 13	19 20	96 39	32 31

* “Hieroglyphic turtle” is a common name, not a scientific designation.

**Table 3:** Potentially problematic freshwater turtle species available in the pet trade in Poland, along with species characteristics likely to influence the risk of abandonment.

Species	Carapace Length of Adult Turtle (cm)	Lifespan (Years)	Minimum Enclosure Size	Basking Temp (°C)	Water	Lighting	Average Cost of a Turtle (USD) *	Price per Set (USD) **	Average Cost per Month (USD) ***	Average Cost per Year (USD) ***
*T. scripta* (IAS)	15–35	30–40	100 × 60 × 45 cm, ≥380 L	26–37	24–26 °C, strong filtration	UVB, heat lamp, 12–14 h	92.5	320	30	360
*Pseudemys* spp.	23–43	30–40	200 × 120 × 45 cm, 300–400 L	28–36	25–28 °C, strong filtration	UVB, heat lamp, 12–13 h	55	450	30	360
*G. pseudogeographica* (IAS)	15–30	>30	100 × 50 × 50 cm, ≥115 L	27–32	22–25 °C, strong filtration	UVB, heat lamp, 10–14 h	21.5	320	35	480
*M. reevesii*	11–24	>24	80 × 40 × 40 cm, 100–150 L	32–35	20–26 °C, strong filtration	UVB, heat lamp, 12–14 h	53	180	30	360

* excluding group and adoption offers. ** for Polish market prices; contains aquarium, filtering equipment, and lamps. *** average cost of maintaining an adult per month (USD); contains electricity and feeding.

## Data Availability

The data presented in this study are available on request from the corresponding author.
